# Association of Tumor Hydroxyindole O-Methyltransferase and Serum 5-Methoxytryptophan with Long-Term Survival of Hepatocellular Carcinoma

**DOI:** 10.3390/cancers13215311

**Published:** 2021-10-22

**Authors:** Bor-Sheng Ko, Shu-Man Liang, Tzu-Ching Chang, Jing-Yiing Wu, Po-Hsun Lee, Yu-Juei Hsu, Cheng-Chin Kuo, Jun-Yang Liou, Kenneth K Wu

**Affiliations:** 1 Department of Internal Medicine, National Taiwan University Hospital, Taipei 100, Taiwan; bskomd@ntu.edu.tw; 2 Department of Hematological Oncology, National Taiwan University Cancer Center, Taipei 100, Taiwan; 3 Institute of Cellular and System Medicine, National Health Research Institutes, Zhunan Town 350, Taiwan; shu-man@nhri.edu.tw (S.-M.L.); ctching9@nhri.edu.tw (T.-C.C.); jywu@nhri.edu.tw (J.-Y.W.); pohsun0530@nhri.edu.tw (P.-H.L.); kuocc@nhri.edu.tw (C.-C.K.); 4 Division of Nephrology, National Defense Medical Center, Department of Medicine Tri-Service General Hospital, Taipei 114, Taiwan; yujuei@mail2000.com.tw; 5 Department of Internal Medicine, College of Medicine, National Taiwan University, Taipei 100, Taiwan; 6 Institute of Biotechnology, College of Life Science, National Tsing-Hua University, Hsinchu 300, Taiwan

**Keywords:** hepatocellular carcinoma, 5-methoxytryptophan, hydroxyindole O-methyltransferase, kynurenine, biomarker

## Abstract

**Simple Summary:**

5-methoxytryptophan (5-MTP) is a tryptophan (Trp) metabolite synthesized by hydroxyindole O-methyltransferase (HIOMT). Expression of HIOMT is decreased in various tumors. However, whether HIOMT expression and serum 5-MTP concentration associate with prognosis of hepatocellular carcinoma (HCC) remains unclear. The aim of this study was to analyze HCC tissue HIOMT mRNA and serum 5-MTP and determine their association with survival following therapeutic liver resection. We found a significant association of serum 5-MTP or tissue HIOMT and serum kynurenine (Kyn) with overall and relapse free (RF) survival of HCC. The combination of serum 5-MTP and Kyn is a potential prognostic biomarker of HCC.

**Abstract:**

5-methoxytryptophan (5-MTP) is a recently discovered tryptophan (Trp) metabolite with anti-inflammatory and tumor-suppressing actions. Its synthesis is catalyzed by hydroxyindole O-methyltransferase (HIOMT). HIOMT levels were reported to be decreased in some patients with colorectal, pancreatic and breast cancer. It is unclear whether tissue HIOMT levels is altered in hepatocellular carcinoma (HCC). It is also unclear whether serum 5-MTP concentration is influenced by HCC. In this study, 150 HCC and adjacent normal liver tissues and serum samples were obtained from the HCC biobank established by a prospective multicenter study. Serum samples from 47 healthy subjects were included as a reference. HIOMT mRNA was measured by real time PCR. Serum 5-MTP and selected Trp metabolites were analyzed by quantitative LC-MS. HCC tissue HIOMT mRNA levels adjusted for adjacent normal tissue HIOMT mRNA levels was associated with overall and relapse-free (RF) survival. Combined serum 5-MTP or tissue HIOMT mRNA and serum kynurenine (Kyn) analysis predicted prolonged overall and RF survival following liver resection. A high serum 5-MTP or tissue HIOMT mRNA and low serum Kyn is associated with long-term survival. In conclusion, tumor tissue HIOMT mRNA and serum 5-MTP are potential biomarkers of HCC, especially when analyzed in combination with serum Kyn.

## 1. Introduction

Hepatocellular carcinoma (HCC) is one of the most lethal malignancies with high morbidity and mortality [1]. Early diagnosed HCC can be treated with tumor resection (therapeutic hepatectomy). However, the relapse rate is high [2]. Response to therapeutic hepatectomy is variable due to the heterogeneity of HCC [3,4]. Multiple approaches are taken to stratify HCC based on metabolic network, gene expression profiling and acetone utilization [4,5]. Those studies suggest that metabolic and genetic profiling are useful in classification of HCC. However, neither global transcriptional nor metabolic analysis provides a link to prognosis of HCC. Recent reports implicate kynurenine (Kyn) and its synthetic enzymes, indoleamine 2, 3 dioxygenase (IDO) as prognostic biomarkers of HCC [6]. It is unknown whether other tryptophan metabolites are associated with the prognosis of HCC.

Intracellular L-tryptophan (Trp) is catabolized to several metabolites in a cell-selective manner. Some bioactive metabolites, notably Kyn, serotonin and melatonin were recognized to possess cancer-regulatory actions [7,8,9,10]. 5-methoxytryptophan (5-MTP), a new member of Trp metabolites, was reported to be a cyclooxygenase-2 (COX-2) suppressing factor, and was named cytoguardin [11,12]. It inhibits A549 cancer cell migration, invasion and epithelial mesenchymal transition and attenuates cancer growth and lung metastasis in a murine xenograft tumor model [13,14]. 5-MTP is produced in human vascular endothelial cells, smooth muscle cells and fibroblasts [15]. Its synthesis is catalyzed by tryptophan hydroxylase-1 (TPH-1) which converts Trp to 5-hydroxytryphan (5-HTP) and hydroxyindole O-methyltransferase (HIOMT), which converts 5-HTP to 5-MTP [13]. HIOMT (also known as acetylserotonin methyltransferase, ASMT) is encoded by a single gene. Three mRNA isoforms are expressed in pineal tissues as a result of alternative splicing [16,17]. Full-length ASMT codes for a 373-amino acid (aa) protein (isoform 373) while exon 6-spliced isoform codes for a 345 aa protein (isoform 345). Exon 6 and 7-spliced isoform codes for a 298-aa protein (isoform 298) [16,17]. Isoform 345 was reported to be the functional enzyme for melatonin biosynthesis [18]. By contract, human ECs and fibroblasts express a truncated HIOMT isoform with sequence identify to ASMT298 (HIOMT298) [19]. Cancer cells also express HIOMT298, albeit at a much lower level than normal cells. Transfection of A549 cells with HIOMT298 or HIOMT373 restores HIOMT catalytic activity and 5-MTP production [19]. Analysis of HIOMT expression in human colorectal, pancreatic and breast cancer tissues reveal reduced HIOMT expression in a majority of cancer tissues [19]. Whether HIOMT acts as a clinical biomarker in any cancer type has not been reported. HIOMT expression in HCC has not been reported. The purpose of this study is to determine whether HCC tissue HIOMT levels may be a cancer biomarker. HCC was selected for this study because of availability of HCC tissues from a prospective multi-center HCC longitudinal study organized by the Taiwan Liver Cancer Network (TLCN) [20]. Serum samples were also available for analysis of 5-MTP and other Trp metabolites including Kyn, serotonin, melatonin, 5-HTP and Trp. The results reveal that tumor HIOMT mRNA and serum 5-MTP in combination with serum Kyn are correlated with long-term survival of HCC following therapeutic hepatectomy.

## 2. Materials and Methods

### 2.1. Patients and Specimens

Pathological specimens and corresponding serum samples of 150 HCC patients were obtained from a biobank established by a prospective multi-center HCC longitudinal study organized by Taiwan Liver Cancer Network (TLCN) [20]. HCC patients eligible for liver resection therapy were recruited to participate in this study starting in 2005. Each patient underwent detailed examinations and the clinicopathological characters as shown in Table 1 were provided by the Biobank located at National Health Research Institutes (NHRI), Taiwan. Patients were followed annually. The median follow-up time is 114.7 months. Study design of the current study, along with the policy of waiving informed consent, was approved by the Institutional Review Board of NHRI and Tri-Service General Hospital (IRB numbers EC1070109-E, 1-104-05-133 and 2-104-05-117).

### 2.2. Quantitative Real-Time PCR (qPCR)

The mRNA extracted from HCC tissues was received from the TLCN. The cellular characteristics of HCC and adjacent liver tissues, as well as quality of the extracted mRNA were confirmed and validated by TLCN. mRNA was extracted from 150-paired HCC tissues and adjacent normal liver tissues and cDNA was synthesized from mRNA using the PrimeScript RT reagent kit (TAKARA, Japan) with oligo-dT and random hexamer primers. Real-time qPCR analysis was performed with SYBR Green (Kapa biosystem, Woburn, MA, USA) using specific oligonucleotide primers of HIOMT (forward, 5′-CAGGAGGTCTGGAGCGTCAA-3′; reverse, 5′-CCTTGCGATAGTTTGCTGAG-3′). Applied Biosystems Relative Quantification Manager Software was used to analyze the relative gene expression by the comparative cycle threshold (Ct) method. HIOMT gene expression was normalized to that of glyceraldehyde-3- phosphate dehydrogenase (GAPDH, forward primer, 5′-CGCTCTCTGCTCCTCCTGTT-3′; reverse, 5′-CCATGGTGTCTGAGCGATGT-3′). HIOMT mRNA in normal (N) and tumor (T) tissues were expressed as 2 − ΔCt × 103 (ΔCt = Ct of HIOMT-Ct of GADPH). The ratio of tumor to normal HIOMT mRNA (T/N ratio) was represented as log2 2-ΔΔCt, where ΔΔCt = ΔCt of tumor-ΔCt of normal. The HIOMT mRNA in normal and tumor tissues and T/N ratio were collected and applied to the survival rate analysis.

### 2.3. Measurement of Trp Metabolites by High Performance Liquid Chromatography-Mass Spectrometry (HPLC-MS)

Tryptophan metabolites including Trp, Kyn, 5-HTP, 5-MTP, melatonin, and serotonin were measured using HPLC coupled with a Q-Exactive Orbitrap Plus mass spectrometer (Thermo Fisher Scientific, Bremen, Germany). Liquid chromatography was performed on HPLC system using a BEH C18 column (1.7 μM, 2.1 mm × 100 mm, Waters Corporation). HPLC linear gradient conditions were: 0–0.5 min 5% B, 0.5–4 min from 5% B to 95% B, 4–5.5 min 95% B, 5.5–5.6 min from 95% B, and 5.6–9 min 5% B [solvent system A: water/formic acid (100:0.1, *v*/*v*); B: acetonitrile/formic acid (100:0.1, *v*/*v*)]. The injection volume was 2 μL, and the column temperature was maintained at 35 °C. Mass spectrometry detection was performed by using a Q-Exactive Orbitrap Plus MS (Thermo Fisher Scientific, Bremen, Germany) equipped with an electrospray ionization (ESI) source operating in positive ionization mode. The online MS analysis was at the Multiple Reaction Monitoring (MRM) mode. The parent ion and daughter ion m/z for Try metabolites were previously described [19]. Quantification of Trp metabolites was done using TraceFinder 4.1 software (Thermo Fisher Scientific, Bremen, Germany). The calibration curves were established by using pure Trp at concentrations of 0.0–47.8 μM, Kyn at concentrations of 0.0–45.7 μM, 5-HTP at concentrations of 0.0–0.45 μM, 5-MTP at concentrations of 0.0–0.4 μM, melatonin at concentrations of 0.0–3.4 nM, serotonin at concentrations of 0.0–4.5 μM, respectively.

### 2.4. Statistical Analysis

A Kruskal-Wallis one-way ANOVA test, and post-hoc Dunnett’s tests (if needed), were utilized to compare the HIOMT expression and metabolite serum level between different clinic-pathological parameters. To correlate the distribution of different metabolites in patient samples or compare expression levels of HIOMT in normal or tumor tissues, a Pearson’s correlation co-efficient (R) was calculated and 2-axis dot plots were drawn. Kaplan-Meier curves were plotted, and the log-rank tests for overall comparison were performed to analyze the time-related probabilities of overall survival and relapse-free survival. All statistical analyses were performed with IBM SPSS Statistics 23 for Windows. Two-side *p* < 0.05 is considered as statistically significant.

## 3. Results

### 3.1. HIOMT mRNA Expression in HCC Tissues Is Correlated with Survival

**Figure 1:**
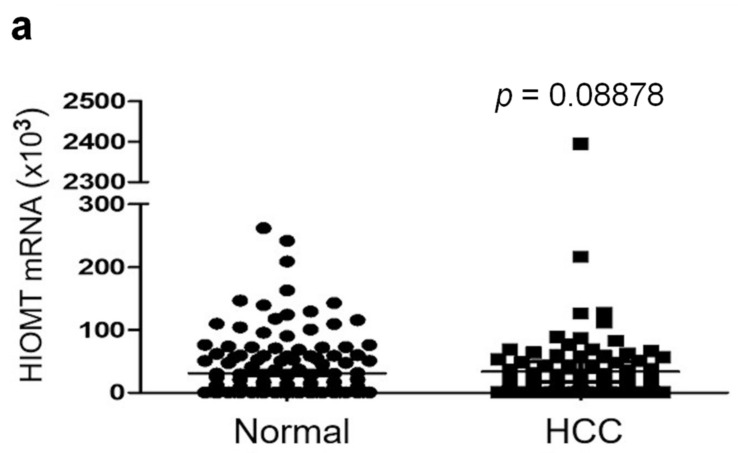

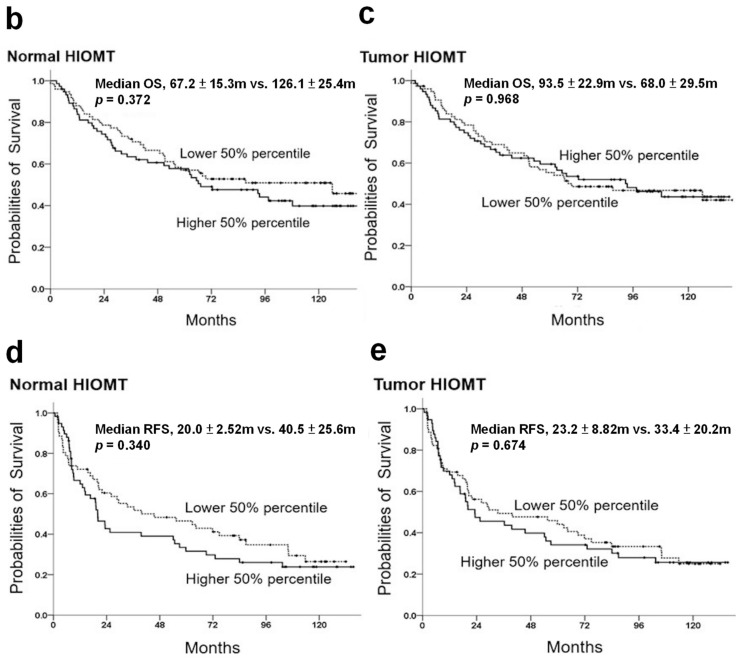
HCC tissue HIOMT mRNA levels and survival. (**a**). Scatter plots of HIOMT mRNA levels measured by qPCR. Normal denotes healthy donors (*n* = 47) and HCC, hepatocellular cancer patients (*n* = 150). (**b**–**e**). Analysis of association of HIOMT mRNA levels with survival by Kaplan-Meier Curve. (**b**) & (**c**). Overall survival (OS) and (**d**) & (**e**). Relapse-free survival (RFS).

**Figure 2:**
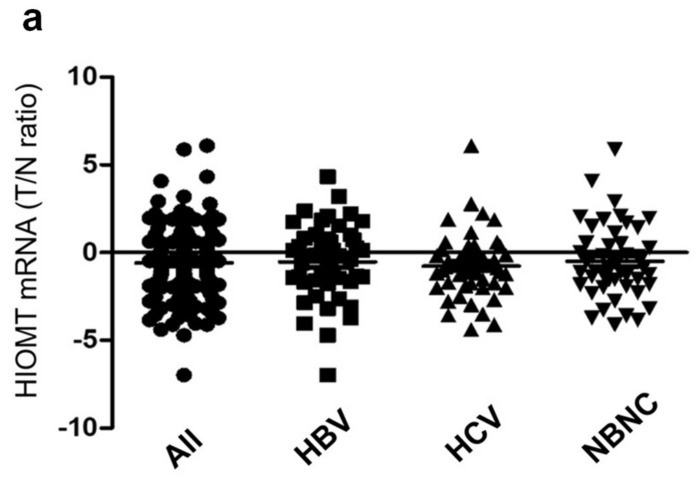

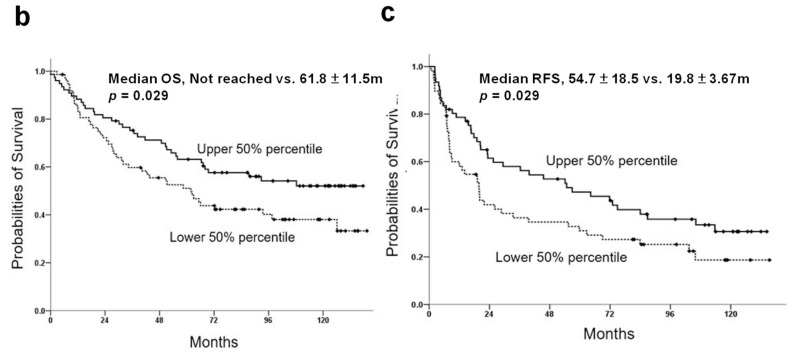
Correlation of tumor/normal tissue HIOMT mRNA levels (T/N) with survival. (**a**). Scatter plots. All denotes entire group of HCC (*n* = 150). HBV, hepatitis B virus, HCV hepatitis C virus and NBNC non-HBV, non-HCV (*n* = 50, each subgroup). (**b**,**c**). Analysis of correlation between T/N value and survival; (**b**). Overall survival (OS) and (**c**). Relapse-free survival (RFS).

HIOMT occupies a pivotal position in 5-MTP synthesis. As 5-MTP controls cancer cell proliferation and migration, we proposed that HIOMT expression in HCC tissues is linked to HCC growth and mortality. HIOMT mRNA levels in 150 paired HCC and adjacent normal tissues were measured by real time PCR (qPCR). As it is not feasible to design primers to identify and measure HIOMT298 selectively, we designed primers which were expected to capture all three isoforms of HIOMT mRNA. HIOMT mRNA levels in HCC tissues were lower than those in normal tissues, although the difference between HCC and normal tissues was not statistically significant (*p* = 0.089) (shown in Figure 1a). HIOMT mRNA levels were higher in patients with hepatitis C viral (HCV) infection (shown in Figure 1b). In this subgroup of HCC, HIOMT level in HCC tissues was significantly lower than that in normal tissues (*n* = 50, *p* < 0.005) (shown in Figure 1b). Association of HIOMT mRNA levels with clinicopathological features of 150 HCC patients is shown in Table 1. HIOMT mRNA level was lower in patients with large tumor (>5 cm), cancer with vascular invasion, advanced cancer stages, and metastasis. However, the differences did not reach statistical significance. Correlation of tissue HIOMT mRNA levels with long-term survival was analyzed by the Kaplan Meier survival curve. Neither normal nor tumor tissue HIOMT mRNA levels were significantly correlated with overall survival (shown in Figure 1b,c) or relapse-free (RF) survival (shown in Figure 1d,e). Of note, the cancer tissue HIOMT mRNA level was positively correlated with HIOMT mRNA levels of paired normal tissues (shown in Figure 2). The relationship between the ratio of cancer tissue mRNA to normal tissue mRNA (T/N) and survival was analyzed. T/N ratio of the entire group of HCC (*n* = 150) and the subgroups (*n* = 50 each subgroup) is shown in Figure 2a. Distribution of T/N ratio of each subgroup is comparable to that of the entire group. After adjusting with normal tissue HIOMT mRNA, tumor HIOMT mRNA in patients with HCV infection was no longer elevated. Importantly, patients with T/N ratio at the upper 50th percentile had a longer overall survival (shown in Figure 2b) and RF survival (shown in Figure 2c) than those at the lower 50th percentile.

### 3.2. Serum Trp Metabolites in HCC vs. Healthy Subjects

**Figure 3:**
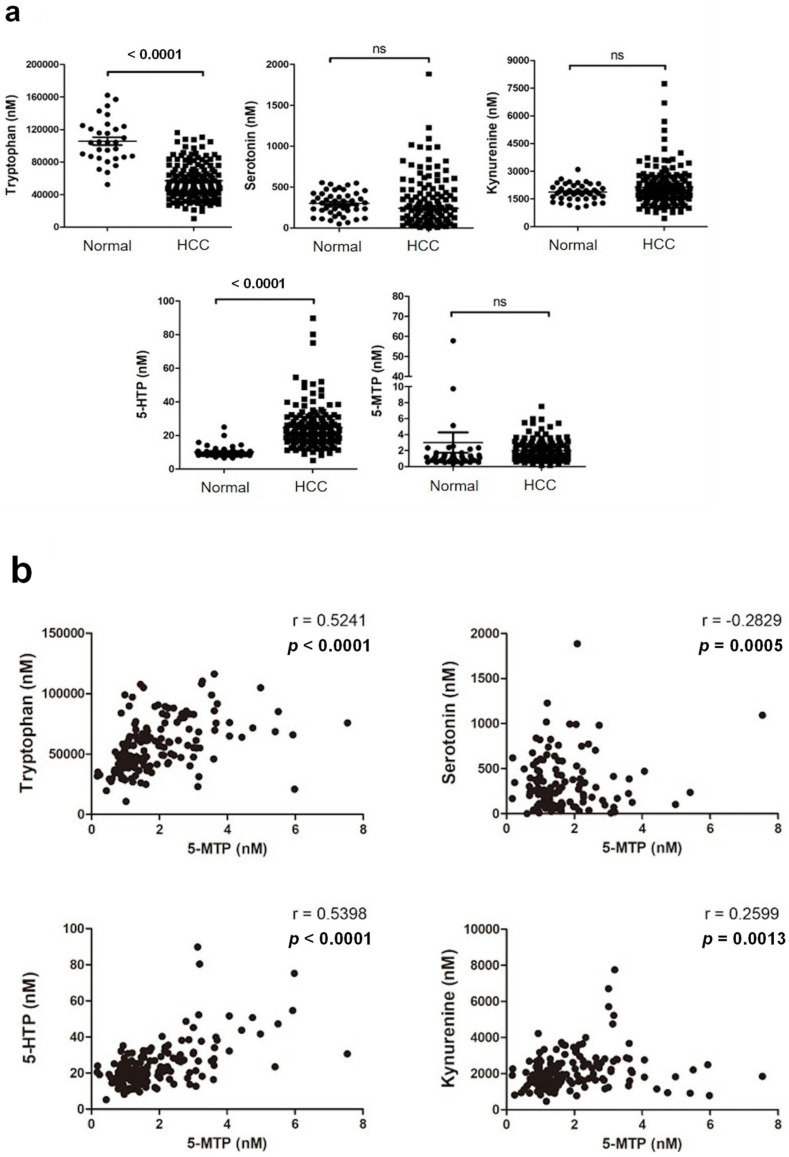
Serum Trp metabolites and survival. (**a**). Scatter plots of serum Trp, serotonin, Kyn, 5-HTP and 5-MTP values of HCC patients (*n* = 150) and healthy subjects (*n* = 47). NS denotes non-significant. (**b**). Correlation between serum Trp metabolite values.

5-MTP and a selected group of Trp metabolites including Trp, 5-hydroxytryptophan (5-HTP), 5-hydroxytryptamine (5-HT, serotonin), melatonin and Kyn in serum samples of 150 HCC patients collected at the time of therapeutic hepatectomy were analyzed by LC-tandem MS. Sera from 47 healthy subjects were included as a reference. Serum 5-MTP levels of HCC patients were not significantly different from that of healthy subjects (shown in Figure 3a). Serum 5-MTP values in HCC patients show a large standard deviation (shown in Table S1) ranging from 0.17 to 7.54 nM. Other bioactive metabolites i.e., serotonin and Kyn in HCC patients were not different from those in healthy subjects (shown in Figure 3a). Serum melatonin was below the detection limit of the assay (shown in Table S1). By contrast, serum Trp level was reduced and 5-HTP level was increased in cancer patients when compared to those in healthy subjects (shown in Figure 3a). The range of serum Trp (10.78–116.32 μM) and 5-HTP (5.19–89.82 μM) was wide, consistent with large variations of the serum values among HCC patients (shown in Table S1). These results suggest that Trp is consumed in HCC for production of Kyn via the IDO/TDO pathway and 5-HTP and its downstream metabolites via the TPH pathway. Serum 5-MTP was positively correlated with Trp and 5-HTP and negatively correlated with serotonin (shown in Figure 3b). Surprisingly, serum 5-MTP was positively correlated with serum Kyn (shown in Figure 3b). These results suggest that HCC influences serum Trp metabolites by a complex regulatory mechanism involving alteration of IDO/TDO and TPH pathways.

We next analyzed correlation of tumor HIOMT mRNA levels with serum 5-MTP concentrations. There was a weak correlation between tumor HIOMT and serum 5-MTP (r = 0.142). However, the correlation was not statistically significant (*p* = 0.083).

### 3.3. Correlation of Serum Metabolites with Clinicopathological Features

Serum 5-MTP concentrations were significantly lower in cigarette smokers and alcohol drinkers (shown in Table 2). Other metabolites were not affected by these two lifestyle habits. Serum 5-MTP was higher in HCC patients with HBV or HCV infections. Similarly, serum 5-HTP, serotonin and Kyn were increased in HCC with viral infections. Serum 5-MTP was low in HCC patients with large tumors (>5 cm) compared to patients with smaller tumors, but the difference was not statistically different (shown in Table 2). Serum 5-MTP was also lower in HCC with capsular vein invasion. Of note, serum 5-MTP was not influenced by age or gender while serum Kyn increased with aging and was higher in males than in females (shown in Table 2).

### 3.4. Association of Metabolite Levels with Long-Term Survival

**Figure 4:**
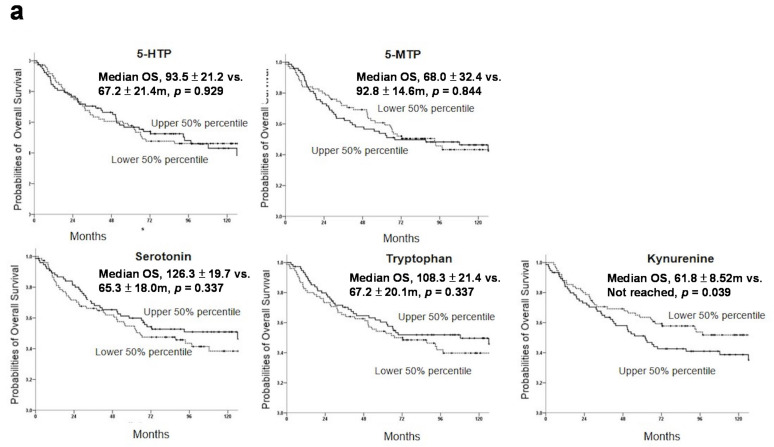

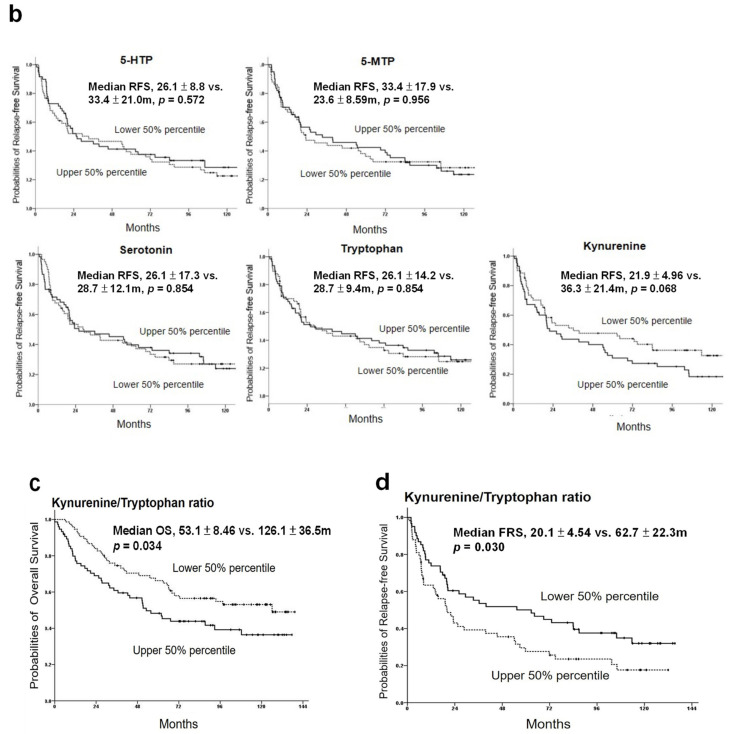
Association of serum Trp metabolite values with survival. (**a**). Overall survival (OS). (**b**). Relapse-free survival (RFS). (**c**) & (**d**). Correlation of Kyn/Trp ratio with (**c**). OS and (**d**). RFS.

As the participants had a long follow-up period (median follow-up was 114.7 months), we were able to determine whether serum Trp metabolites are associated with long-term survival. Serum 5-MTP was not associated with overall of RF survival (shown in Figure 4a,b). Similarly, neither serotonin nor 5-HTP or Try was associated with survival (shown in Figure 4a,b). By contrast, serum Kyn levels were significantly associated with overall survival (shown in Figure 4a). The Kyn values at the upper 50th percentile had a significantly lower survival than that at the lower survival (61.8 ± 8.52 months vs. > 120 months) (shown in Figure 4a). Kyn level was also associated with RF survival (shown in Figure 4b). Kyn/Trp ratio was used as a surrogate marker of IDO-1. We analyzed the association of Kyn/Trp ratio with HCC survival. Kyn/Trp ratio at the lower 50th percentile had a significantly longer overall and RF survival than that at the upper 50th percentile (shown in Figure 4c,d). Compared to individual Kyn analysis, the Kyn/Trp ratio provided a better discrimination of overall and RF survival.

### 3.5. Analysis of Survival Correlations by Combining Kyn with 5-MTP or HIOMT

**Figure 5:**
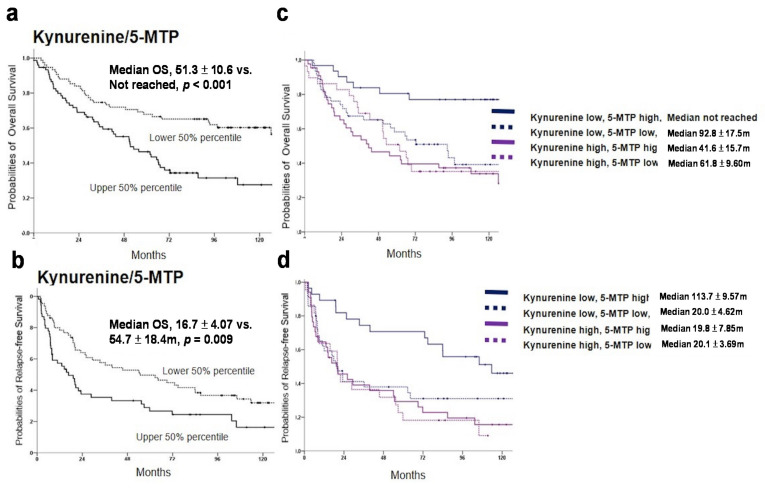

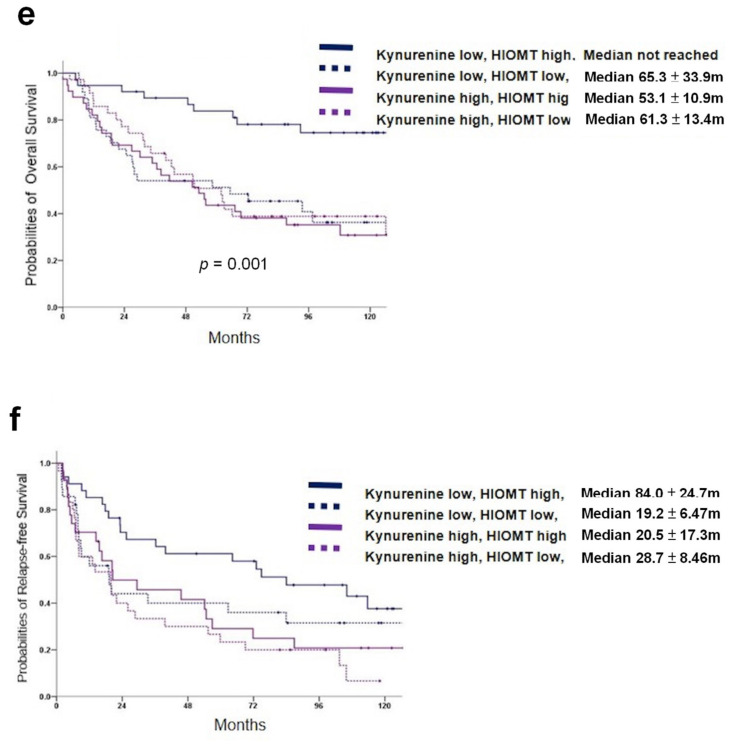
Analysis of survival association by Kyn and 5-MTP or HIOMT combination analysis. (**a**) & (**b**). Association of Kyn/5-MTP ratio with (**a**). OS and (**b**). RFS. (**c**) & (**d**). Association of Kyn plus 5-MTP analysis with (**c**). OS and (**d**). RFS. Kyn or 5-MTP low and Kyn or 5-MTP high denote serum Kyn or 5-MTP concentrations at lower 50th and upper 50th percentiles, respectively. (**e**) & (**f**). Association of Kyn plus HIOMT analysis with (**e**). OS and (**f**). RFS. HIOMT high and low denote HCC tissue HIOMT mRNA levels at upper 50th and lower 50th percentiles, respectively. The cutoff value for serum Kyn and 5-MTP are 1910 nM and 1.54 nM. The cutoff level of T/N ratio for HIOMT is 0.847.

As Kyn and 5-MTP possess opposite effects on cancer growth, we determined the association of Kyn/5-MTP ratio with HCC clinicopathological features and survival. A high ratio was associated with older age, alcohol drinking and cigarette smoking (shown in Table S2). A Kyn/5-MTP ratio at the upper 50th percentile was associated with a short overall survival when compared to that at the lower 50th percentile (median 51.3 ± 10.5 months vs. >120 months) (shown in Figure 5a). Kyn/5-MTP ratio provides a more clear separation of survival prediction between high and low values. Kyn/5-MTP at the upper 50th percentile also had a shorter survival than Kyn/5-MTP at the lower 50th percentile but the difference in RF survival between these two groups is not as distinct as that in overall survival (shown in Figure 5b). We next determined whether individuals with a low Kyn and a high 5-MTP have a survival advantage. Individuals with low Kyn (lower 50th percentile) and high 5-MTP (upper 50th percentile) had a very long overall survival (shown in Figure 5c). High Kyn + high 5-MTP had an overall survival curve similar to high Kyn + low 5-MTP or low Kyn + low 5-MTP (shown in Figure 5c). A low Kyn and high 5-MTP confers a long RF survival (median survival: 113.7 ± 9.57 months) (shown in Figure 5d). These results suggest that a low serum Kyn + a high serum 5-MTP is predictive of very long survival and possible cure after therapeutic hepatectomy for HCC.

Combined serum Kyn and tissue HIOMT mRNA analysis predicted prolonged survival in a trend analogous to Kyn + 5-MTP combined analysis (shown in Figure 5e). Individuals with low Kyn and high HIOMT had >80% chance of overall survival over 10 years. They had a long median RF survival (84.0 ± 24.7 months) when compared to RF survival of high T/N HIOMT mRNA (54.7 ± 18.5 months) or low Kyn (36.3 ± 21.4 months) alone (shown in Figure 5f). These results suggest that combined Kyn/5-MTP or Kyn/HIOMT analysis is useful in identifying long survival after therapeutic hepatectomy.

## 4. Discussion

A novel finding of this study is that tumor HIOMT predicts HCC patient long-term survival following therapeutic hepatectomy. When adjusted for adjacent normal tissue HIOMT expression, a high tumor HIOMT mRNA level is associated with an > 10 year overall survival. A high (upper 50th percentile) tumor/normal tissue HIOMT ratio also predicts a prolonged RF survival. It is unclear why tumor tissue or normal tissue HIOMT mRNA levels per se is not correlated with survival. As there exists a positive correlation between tumor and normal HIOMT expression, it is possible that a high ratio reflects a relatively high tumor HIOMT expression and a consequent high 5-MTP in tumor microenvironment to control cancer progression. Several cell types in normal liver and HCC tissues are potential sources of HIOMT expression and 5-MTP production. Vascular ECs and foreskin fibroblasts express HIOMT298 and produce 5-MTP at the basal state [13,15,19]. It is likely that hepatic vascular ECs and hepatic stellate cells express HIOMT and produce 5-MTP. It is unclear whether hepatocytes express 5-MTP producing enzymes. HCC cells such as HepG2 and Huh7 cells, like A549 cells, express only low levels of HIOMT298 and produce subnormal 5-MTP. Transfection of A549 and other cancer cells including Huh7 cells with HIOMT298 results in restoring 5-MTP production accompanied by reduction of cancer cell malignant phenotype [19]. 5-MTP suppresses cancer cell COX-2 and MMP-9 expression and inhibits cancer cell migration and invasion [19]. Administration of 5-MTP in a murine xenograft tumor model reduces lung metastasis [13]. Implantation of HIOMT298-transfected A549 cells in the xenograft tumor model shows reduced lung metastasis [19]. Taken together, these findings suggest that a high HIOMT expression in HCC tissue relative to normal tissue confers prolonged survival through 5-MTP-mediated control of cancer metastasis.

HCC influences serum Trp concentrations. Consistent with reported decrease of serum Trp in colorectal and ovarian cancer [21,22], our results show that the mean serum Trp concentration in HCC patients is significantly lower than that of healthy subjects. Reduced serum Trp concentration in cancer was considered to be due to Trp catabolism to generate Kyn via IDO/TDO [23]. Our results suggest that HCC accelerates Trp catabolism via the TPH pathway, resulting in the accumulation of 5-HTP in circulating blood. As the cellular source of circulating 5-HTP is unknown and may not be directly derived from cancer cells, interpretation of serum concentrations of Trp metabolites requires caution. It would be more informative to measure metabolites in the tumor microenvironment.

A number of cancer cells, including HCC cells, express TDO and/or IDO-1, which catalyze Kyn production [7]. Kyn promotes cancer growth by binding to aryl hydrocarbon receptors [7]. In addition, Kyn induces immunosuppressive cells such as regulatory T cells to help cancer evade immunosurveillance [23]. In view of its importance in regulating cancer growth, analysis of serum Kyn and its association with cancer prognosis has been extensively reported. In this study, we analyzed serum Kyn in 150 HCC patients and 47 healthy subjects and did not detect a significant difference between HCC and healthy subjects. Our results are similar to several reports which indicate that serum Kyn level was not elevated in lung or pancreatic cancer [24,25]. On the other hand, serum Kyn was reported to be increased in breast cancer and acute T cell leukemia/lymphoma and reduced in colon cancer [26,27,28]. Different results from those studies may be due to different assays, patient population, Kyn catabolism and cancer heterogenicity. Despite differences in reported serum Kyn values in various cancers, serum Kyn and its catabolite, 3-hydroxyanthranilic acid (HAA), are associated with survival [27,28]. A high serum Kyn is associated with shorter survival than a low serum Kyn. In addition, Kyn/Trp which serves as a surrogate of IDO activity [29] is correlated with HCC survival as reported here as well as with lung, pancreatic cancer and ATLL [25,26,30]. Our results reveal that the Kyn/Trp ratio and Kyn alone had a similar predictive pattern.

It is interesting to note that serum 5-MTP exhibits a negative correlation with serum serotonin. This may be attributed to control of serotonin production by 5-MTP via inhibiting expression of aromatic amino acid decarboxylase (AADC) [19]. AADC catalyzes decarboxylation of 5-HTP to generate 5-hydroxytryptamine (5-HT, serotonin) [31]. Its expression is elevated in carcinoid tumors and neuroendocrine [32,33]. Under the situation of reduced HIOMT expression, cancer cells express AADC and produce abundant serotonin, which promotes cancer progression [8,34]. Transfection of cancer cells with HIOMT restores 5-MTP production, which is accompanied by reduction of AADC expression and the decline of serotonin production [19]. It is possible that HCC with high HIOMT expression may suppress AADC, thereby reducing serotonin release.

Our findings indicate that analysis of survival outcome by combining metabolites with opposite effects on cancer growth enhances the power to identify HCC patients who have prolonged survival following liver resection therapy. Combined Kyn and 5-MTP analysis by taking Kyn/5-MTP ratio has a better discriminatory power than Kyn or Kyn/Trp ratio. An important finding is that HCC patients with a high 5-MTP value and low Kyn value have a very high probability to live longer than 10 years after initial liver resection. That, combined with serum Kyn and tissue HIOMT analysis yields survival data resembling combined Kyn and 5-MTP analysis supports the concept that HIOMT catalyzed 5-MTP production confers protection against cancer progression. The results imply that HCC growth is governed by a balance between tumor-promoting metabolites, notably Kyn, and tumor-suppressing molecules such as 5-MTP. Combined serum Kyn and 5-MTP or serum 5-MTP/tissue represents a new class of prognostic biomarkers for HCC. However, this study has limitations. First, serum Trp metabolite analysis is based on a single blood sample collected prior to liver resection. The study will be strengthened by follow-up Trp metabolites analysis. Longitudinal follow-up analysis will allow for a more precise interpretation of the correlation of serum metabolite values and HCC progression. Second, we were unable to measure tumor 5-MTP and Kyn due to sample restrictions. Direct measurement of those metabolites in cancer tissue will gain insights into the genesis of circulating 5-MTP and Kyn and provide more detailed information about the correlation of Trp metabolites with HCC growth and metastasis.

## 5. Conclusions

Our findings indicated that the combination of serum 5-MTP or tissue HIOMT levels and serum Kyn is a potential prognostic biomarker of HCC.

## Figures and Tables

**Table 1:** Association of HIOMT with clinicopathological features.

Characters	*n*	HIOMT	*p*
Total	150		
Age ≦60 y/o >60 y/o	68 82	−0.53 ± 1.97 −0.65 ± 1.99	NS
Gender Male Female	102 48	−0.76 ± 1.96 −0.24 ± 1.99	NS
Smoking Yes No Unknown	72 75 3	−0.86 ± 1.80 −0.33 ± 2.14	NS
Drinking Yes No Unknown	31 116 3	−0.52 ± 1.77 −0.61 ± 2.54	NS
Tumor size (diameter) ≦5 cm >5 cm	70 80	−0.31 ± 2.12 −0.84 ± 1.82	NS
Pathology type Solitary Multiple Infiltrative	103 46 1	−0.53 ± 2.04 −0.72 ± 1.87 −1.78	NS
Vascular invasion Absent Capsular vein Portal vein invasion	49 18 83	−0.41 ± 1.92 −0.56 ± 2.01 −0.71 ± 2.02	NS
AJCC staging Stage I Stage II Stage III Stage IV	41 57 47 5	−0.66 ± 1.70 −0.32 ± 2.00 −0.82 ± 2.25 −1.13 ± 0.76	NS
BCLC staging Stage A Stage B Stage C	59 58 33	−0.13 ± 1.92 −0.87 ± 1.67 −0.95 ± 2.43	0.065
Cirrhosis Yes No	46 104	−0.53 ± 2.19 −0.63 ± 1.89	NS
Alpha-fetoprotein ≦80 ng/mL >80 ng/mL	92 58	−0.51 ± 1.93 −0.72 ± 2.07	NS
Viral infection Hepatitis B Hepatitis C No hepatitis B or C	50 50 50	−0.52 ± 2.10 −0.77 ± 1.82 −0.50 ± 2.03	NS
Metastasis Yes No	9 141	−1.30 ± 1.30 −0.55 ± 2.01	NS

Abbreviations: AJCC, American Joint Committee on Cancer; BCLC, Barcelona Clinic Liver Cancer.

**Table 2:** Correlation of selected serum Trp metabolites with HCC clinicopathological features.

Characters	*n*	Log_5-MTP	*p*	Log_5-HTP	*p*	Log_TRP	*p*	Log_SER	*p*	Log_KYN	*p*
Total	150										
Age ≤60 y/o >60 y/o	68 82	0.22 ± 0.30 0.19 ± 0.26	NS	1.35 ± 0.19 1.34 ± 0.20	NS	4.76 ± 0.15 4.69 ± 0.19	0.015	2.46 ± 0.46 2.20 ± 0.68	0.019	3.25 ± 0.22 3.31 ± 0.17	0.073
Gender Male Female	102 48	0.21 ± 0.27 0.19 ± 0.29	NS	1.35 ± 0.19 1.34 ± 0.21	NS	4.75 ± 0.18 4.67 ± 0.16	0.020	2.36 ± 0.61 2.23 ± 0.57	NS	3.31 ± 0.18 3.14 ± 0.21	0.040
Smoking Yes No Unknown	72 75 3	0.16 ± 0.27 0.26 ± 0.28	0.032	1.32 ± 0.18 1.38 ± 0.21	NS	4.73 ± 0.16 4.72 ± 0.20	NS	2.36 ± 0.65 2.28 ± 0.53	NS	3.28 ± 0.16 3.29 ± 0.22	NS
Drinking Yes No Unknown	31 116 3	0.11 ± 0.24 0.23 ± 0.28	0.032	1.34 ± 0.20 1.35 ± 0.20	NS	4.71 ± 0.17 4.73 ± 0.18	NS	2.43 ± 0.42 2.30 ± 0.64	NS	3.33 ± 0.18 3.27 ± 0.19	NS
Tumor size (diameter) ≤5 cm >5 cm	70 80	0.24 ± 0.25 0.17 ± 0.30	NS	1.33 ± 0.19 1.36 ± 0.20	NS	4.77 ± 0.15 4.68 ± 0.19	0.004	2.37 ± 0.50 2.28 ± 0.67	NS	3.29 ± 0.16 3.28 ± 0.22	NS
Pathology type Solitary Multiple Infiltrative	103 46 1	0.21 ± 0.26 0.20 ± 0.31	NS	1.33 ± 0.19 1.36 ± 0.20	NS	4.72 ± 0.18 4.74 ± 0.15	NS	2.27 ± 0.67 2.40 ± 0.44	NS	3.29 ± 0.16 3.28 ± 0.22	NS
Vascular invasion Absent Capsular vein Portal vein invasion	49 18 83	0.24 ± 0.24 0.09 ± 0.29 0.20 ± 0.29	NS	1.34 ± 0.19 1.26 ± 0.20 1.37 ± 0.19	0.067	4.74 ± 0.17 4.59 ± 0.20 4.74 ± 0.17	0.002	2.38 ± 0.52 1.87 ± 1.19 2.37 ± 0.44	0.020	3.28 ± 0.19 3.28 ± 0.18 3.29 ± 0.20	NS
AJCC staging Stage I Stage II Stage III Stage IV	41 57 47 5	0.26 ± 0.24 0.14 ± 0.27 0.23 ± 0.31 0.26 ± 0.16	NS	1.35 ± 0.18 1.31 ± 0.21 1.40 ± 0.18 1.31 ± 0.02	0.084	4.75 ± 0.18 4.69 ± 0.17 4.74 ± 0.17 4.82 ± 0.08	NS	2.38 ± 0.55 2.17 ± 0.73 2.46 ± 0.45 2.16 ± 0.19	0.143	3.27 ± 0.19 3.25 ± 0.17 3.32 ± 0.22 3.49 ± 0.12	0.026
BCLC staging Stage A Stage B Stage C	59 58 33	0.26 ± 0.24 0.10 ± 0.30 0.30 ± 0.24	0.001	1.34 ± 0.19 1.32 ± 0.21 1.40 ± 0.16	NS	4.76 ± 0.15 4.66 ± 0.18 4.76 ± 0.18	0.002	2.37 ± 0.56 2.19 ± 0.73 2.47 ± 0.42	NS	3.29 ± 0.17 3.25 ± 0.20 3.33 ± 0.22	NS
Cirrhosis Yes No	46 104	0.24 ± 0.25 0.19 ± 0.29	NS	1.38 ± 0.17 1.34 ± 0.20	NS	4.78 ± 0.15 4.70 ± 0.18	0.011	2.30 ± 0.46 2.33 ± 0.65	NS	3.35 ± 0.18 3.26 ± 0.19	0.012
Alpha- fetoprotein ≤80 ng/mL >80 ng/mL	92 58	0.19 ± 0.27 0.22 ± 0.30	NS	1.34 ± 0.19 1.36 ± 0.21	NS	4.72 ± 0.16 4.72 ± 0.19	NS	2.22 ± 0.66 2.50 ± 0.42	0.015	3.28 ± 0.18 3.29 ± 0.21	NS
Viral infection Hepatitis B Hepatitis C No hepatitis B or C	50 50 50	0.29 ± 0.27 0.20 ± 0.29 0.12 ± 0.25	0.005	1.41 ± 0.19 1.37 ± 0.19 1.26 ± 0.18	< 0.001	4.84 ± 0.15 4.67 ± 0.14 4.64 ± 0.16	< 0.001	2.57 ± 0.39 2.25 ± 0.50 2.12 ± 0.78	0.003	3.34 ± 0.19 3.29 ± 0.20 3.23 ± 0.18	0.026
Metastasis Yes No	9 141	0.24 ± 0.16 0.20 ± 0.28	NS	1.36 ± 0.14 1.35 ± 0.20	NS	4.80 ± 0.09 4.72 ± 0.18	NS	2.33 ± 0.41 2.33 ± 0.61	NS	3.43 ± 0.15 3.28 ± 0.19	0.025

## Data Availability

The data presented in this study are available on request from the corresponding author.

## Supplementary Materials

The following are available online at https://www.mdpi.com/article/10.3390/cancers13215311/s1, Figure S1: Scatter plots of tissue HIOMT mRNA levels according to viral infection status. Figure S2: Correlation of HCC tissue with adjacent normal tissue HIOMT mRNA levels. Table S1: Distribution of serum metabolite values of 150 HCC patients. Table S2: Correlation of Kyn/5-MTP with clinicopathological features.
