# Supplementary material for: Association of Tumor Hydroxyindole O-Methyltransferase and Serum 5-Methoxytryptophan with Long-Term Survival of Hepatocellular Carcinoma
Source: Cancers (Basel). 2021 Oct 22;13(21):5311. doi: 10.3390/cancers13215311 (PMC8582430; doi:10.3390/cancers13215311)
Supplement: Supplementary file 1 [file cancers-13-05311-s001.zip › cancers-1409460-supplementary.pdf]

Association of Tumor Hydroxyindole O-Methyltransferase and Serum 5-Methoxytryptophan with Long-Term Survival of Hepatocellular Carcinoma

Bor-Sheng Ko, Shu-Man Liang, Tzu-Ching Chang, Jing-Yiing Wu, Po-Hsun Lee, Yu-Juei Hsu, Cheng-Chin Kuo, Jun-Yang Liou and Kenneth K Wu

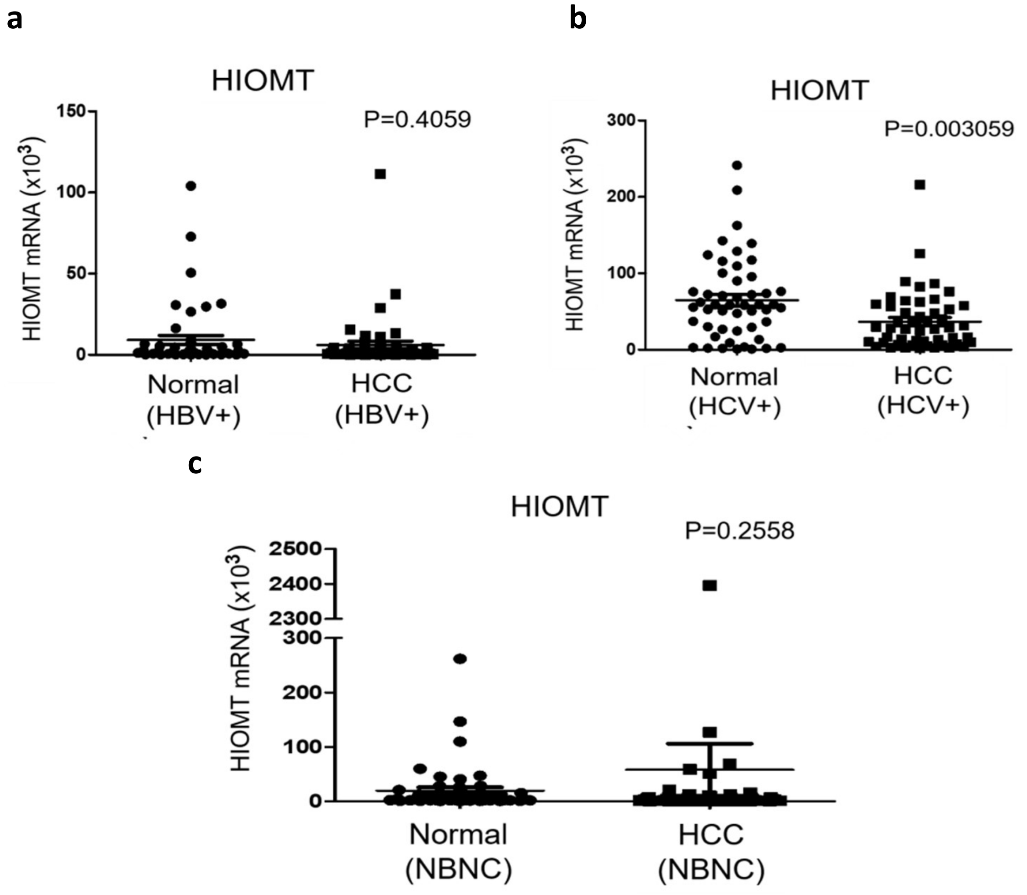

Figure S1. Scatter plots of tissue HIOMT mRNA levels according to viral infection status. (**a**). HBV, (**b**). HCV and (**c**). Non-HBV, non-HCV. Normal denotes adjacent normal tissue (n = 50 each subgroup) and HCC denotes hepatocellular cancer tissue HIOMT mRNA.

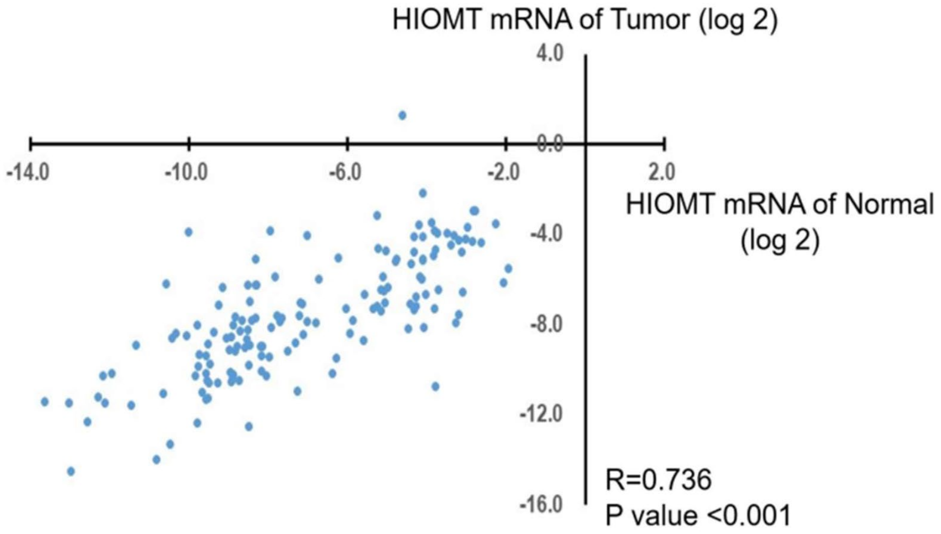

Figure S2. Correlation of HCC tissue with adjacent normal tissue HIOMT mRNA levels. Each dot denotes a paired tissue HIOMT mRNA. Tumor HIOMT mRNA levels are highly correlated with adjacent normal tissue HIOMT mRNA levels (r = 0.736; $p < 0.001$).

Table S1. Distribution of serum metabolite values of 150 HCC patients.

Variation	5-MTP (nm)	Trp (μM)	5-HTP (nM)	5-HT (μM)	MLT (nM)	Kyn (μM)
Mean	1.94	56.97	24.72	0.24	0	2.14
SD	1.25	21.70	12.68	0.33	0	1.06
Median	1.54	53.91	21.75	0.17	0	1.91
25%	1.06	41.75	17.17	0	0	1.52
75%	2.57	70.63	29.40	0.39	0	2.58

Abbreviations: 5-MTP, 5-methoxytryptophan; Trp, tryptophan; 5-HTP, 5-hydroxytryptophan; 5-HT, 5-hydroxytryptamine (serotonin); MLT, melatonin and Kyn, kynurenine.

Table S2. Correlation of Kyn/5-MTP with clinicopathological features.

Characters	*n*	Log (KYN/5-MTP)	*p*
Total	150		
Age			
≦60 y/o	68	3.03 ± 0.32	0.070
＞60 y/o	82	3.12 ± 0.28	
Gender			
Male	102	3.10 ± 0.28	NS
Female	48	3.05 ± 0.34	
Smoking			
Yes	72	3.13 ± 0.29	0.056
No	75	3.03 ± 0.30	
Unknown	3		
Drinking			
Yes	31	3.22 ± 0.18	0.003
No	116	3.04 ± 0.31	
Unknown	3		
Tumor size (diameter)			
≦5 cm	70	3.05 ± 0.27	NS
＞5 cm	80	3.11 ± 0.32	
Pathology type			
Solitary	103	3.07 ± 0.28	
Multiple	46	3.12 ± 0.34	NS
Infiltrative	1		
Vascular invasion			
Absent	49	3.04 ± 0.29	
Capsular vein	18	3.19 ± 0.25	NS
Portal vein invasion	83	3.08 ± 0.31	
AJCC staging			

Stage I	41	3.01 ± 0.31	NS
Stage II	57	3.11 ± 0.24	
Stage III	47	3.09 ± 0.36	
Stage IV	5	3.22 ± 0.20	
BCLC staging			
Stage A	59	3.04 ± 0.26	
Stage B	58	3.15 ± 0.33	0.071
Stage C	33	3.03 ± 0.30	
Cirrhosis			
Yes	46	3.11 ± 0.27	NS
No	104	3.07 ± 0.31	
Alpha-fetoprotein			
≦80 ng/ml	92	3.09 ± 0.31	NS
＞80 ng/ml	58	3.07 ± 0.29	
Viral infection			
Hepatitis B	50	3.04 ± 0.30	
Hepatitis C	50	3.08 ± 0.32	NS
No hepatitis B or C	50	3.12 ± 0.28	
Metastasis			
Yes	9	3.18 ± 0.17	NS
No	141	3.08 ± 0.31	
